# Selenoprotein P Is the Major Selenium Transport Protein in Mouse Milk

**DOI:** 10.1371/journal.pone.0103486

**Published:** 2014-07-28

**Authors:** Kristina E. Hill, Amy K. Motley, Virginia P. Winfrey, Raymond F. Burk

**Affiliations:** Division of Gastroenterology, Hepatology, and Nutrition, Department of Medicine, Vanderbilt University School of Medicine, Nashville, Tennessee, United States of America; National Institute for Research in Reproductive Health, India

## Abstract

Selenium is transferred from the mouse dam to its neonate via milk. Milk contains selenium in selenoprotein form as selenoprotein P (Sepp1) and glutathione peroxidase-3 (Gpx3) as well as in non-specific protein form as selenomethionine. Selenium is also present in milk in uncharacterized small-molecule form. We eliminated selenomethionine from the mice in these experiments by feeding a diet that contained sodium selenite as the source of selenium. Selenium-replete dams with deletion of *Sepp1* or *Gpx3* were studied to assess the effects of these genes on selenium transfer to the neonate. *Sepp1* knockout caused a drop in milk selenium to 27% of the value in wild-type milk and a drop in selenium acquisition by the neonates to 35%. In addition to decreasing milk selenium by eliminating Sepp1, deletion of *Sepp1* causes a decline in whole-body selenium, which likely also contributes to the decreased transfer of selenium to the neonate. Deletion of *Gpx3* did not decrease milk selenium content or neonate selenium acquisition by measurable amounts. Thus, when the dam is fed selenium-adequate diet (0.25 mg selenium/kg diet), milk Sepp1 transfers a large amount of selenium to neonates but the transfer of selenium by Gpx3 is below detection by our methods.

## Introduction

Selenium was reported to be a constituent of milk in 1935 [1] and was recognized to be present in milk protein in 1948 [2]. Picciano and colleagues carried out studies in animal and human milk in the 1980s and reported that the selenoprotein glutathione peroxidase (Gpx) was present in milk and that as much as 30% of milk selenium was dialyzable [3], [4], [5]. Avissar and colleagues later identified human milk Gpx to be Gpx3, the major Gpx form present in plasma, and showed that it accounted for less than 15% of the selenium in human milk [6]. More recently, Schweizer and colleagues presented results indicating that selenoprotein P (Sepp1) is present in mouse milk [7]. Thus, the two plasma selenoproteins, Sepp1 and Gpx3, are present in milk, as are dialyzable forms of selenium.

Many proteins containing selenomethionine–termed selenium-containing proteins–are present in the body and presumably also are present in milk. Selenomethionine is synthesized by plants and incorporated into their proteins. It is a major dietary source of selenium for free-living animals. The amount of selenium consumed in the form of selenomethionine is highly variable and depends largely on the geographic source of the diet. Once ingested, selenomethionine enters the methionine metabolic pool [8] and is incorporated randomly into proteins at methionine positions. Only when selenomethionine is catabolized is selenium released from it and recognized as selenium in the animal. For that reason, studies seeking to elucidate specific selenium metabolic processes–such as the study reported here–usually feed selenium in an inorganic form, which enters the selenium metabolic pool directly. Otherwise measurements of selenium would reflect both the specific and the highly variable non-specific forms of selenium and render results difficult to correlate with specific selenium metabolic processes.

In recent years, mice with deletion of *Sepp1* and *Gpx3* have been used to study selenium metabolism. Sepp1 is a selenium-rich protein that transports the element from the liver to peripheral tissues [9]. Apolipoprotein E receptor-2 (apoER2) on cells in those tissues binds Sepp1 arriving via the systemic circulation and facilitates its endocytosis [10]. Sepp1 accounts for about two-thirds of plasma selenium in selenium-replete mice. Although it contains up to one-third of mouse plasma selenium, Gpx3 does not appear to have a selenium transport role except in the visceral yolk sac [11]. Small molecule forms account for less than 3% of plasma selenium [12].

The present study used mice with deletion of the genes for Sepp1 and Gpx3 to assess transfer of selenium from the nursing dam to the neonate by these selenoproteins. The results indicate that Sepp1 is important for this transport in selenium-replete mice but that Gpx3 is not.

## Materials and Methods

### Animals

Because male Sepp1^−/−^ mice are sterile, Sepp1^+/−^ breeding pairs in our colony were mated to produce Sepp1^−/−^ and Sepp1^+/+^ mice for study [13]. Homozygote and wild-type breeding pairs produced the Gpx3^−/−^ and Gpx3^+/+^ female mice [12]. To produce mice with deletion of both Sepp1 and Gpx3, Sepp1^−/−^ female mice were mated with Gpx3^−/−^ male mice. The resulting mice were heterozygotes for both genes and female progeny were mated with Gpx3^−/−^ male mice to produce breeding pairs of Sepp1^+/−^/Gpx3^−/−^ mice. At weaning Sepp1^−/−^/Gpx3^−/−^ pups were selected for study after their genotype had been verified by PCR [12]. Wild-type mice were C57BL/6 strain.

Weanling mice were fed selenium-deficient Torula yeast-based diet supplemented with 0 or 0.25****mg selenium as sodium selenite per kg diet. The diet was formulated and pelleted to our specifications [14] by Harlan-Teklad (Madison, WI). The mice were housed on aspen shavings and had free access to food and tap water. The light cycle in the room was 14****h on and 10****h off. This study was carried out in strict accordance with the recommendations in the Guide for the Care and Use of Laboratory Animals of the National Institutes of Health. The protocol was approved by the Vanderbilt University Institutional Animal Care and Use Committee (Protocol numbers: M/07/304 and M/10/306). All procedures were performed using isoflurane or sodium pentobarbital anesthesia prior to exsanguination, and all efforts were made to minimize suffering.

### Neonate experiments

Pregnant *Sepp1^−/−^* mice were fed diet containing 0.25 mg selenium/kg. Neonates were killed by isoflurane inhalation immediately after being taken from the dam 1 or 5 days after birth. A 0.5 cm portion of the tail was taken from neonates for isolation of DNA and genotyping. Each neonate was weighed, frozen in liquid N_2_, and stored at −80°C until being assayed for selenium content.

### Milk experiments

Milk was collected from nursing dams between days 10 and 16 of lactation. Neonates were removed from their dam and killed by isoflurane inhalation. Three hours after removal of the neonates, the dam was injected intraperitoneally with 3 units of oxytocin. Thirty to 60 min later, the dam was anesthetized (2 mg pentobarbital intraperitoneally) and milk was collected by application of gentle vacuum to each teat. Following milk collection (0.1–0.3 ml/dam), the dam was killed by isoflurane inhalation. Milk was stored at −20°C until it was assayed for selenium content. Most individual milk samples were assayed alone but samples with low volume were pooled within experimental groups.

### ^75^Se labeling of milk

Ten µCi of ^75^Se-selenite (University of Missouri, Columbia, MO, specific activity ∼1000 Ci/g) was administered to nursing dams intraperitoneally approximately 24 h prior to milk collection. Milk was collected in PBS and centrifuged at 16,000 *g* for 15 min to separate the lipid layer, the aqueous layer (whey), and the solids (curd). The whey fraction was transferred to a clean tube. A sample of whey was subjected to SDS-PAGE and the gel was stained with Coomassie blue. The stained 15% acrylamide gel was dried and exposed to Kodak BioMax XAR film.

### Selenium analysis

Tissues and milk were predigested in concentrated nitric acid prior to perchloric acid digestion. Fluorometric determination of selenium was carried out by the method of Koh and Benson [15] as modified by Sheehan and Gao [16].

### *In situ* hybridization

Freshly harvested mammary tissue was processed for in situ hybridization detection of Sepp1 and Gpx3 mRNA. A 641 bp mSEPP1 cDNA was prepared by PCR using the plasmid MB23A1, which contains a full-length mSEPP1 insert, and primers 5′-AGCCAGCTGATACTTGTGTCTTCTGCAGGCAT-3′ and 5′-AAAGGTGCAAGCCTTCACTTGCTGTGGTGT-3′. The PCR product was gel-purified and ligated into the pCR4 Topo plasmid (Invitrogen, Carlsbad, CA). Following transformation into OneShot Top10 cells (Invitrogen, Carlsbad, CA), individual clones were analyzed by PCR to identify the orientation of the mSEPP1 cDNA insert. Clones with opposite insert orientation were used to prepare template DNA by PCR, which included the plasmid T7 promoter and the mSepp1 insert. The purified PCR products were used in transcription reactions to prepare sense and antisense digoxigenin-labeled riboprobes as described previously [17]. These were used for in situ hybridization on tissue cryosections using our standard protocol [17].

A 448 bp mouse GPX3 cDNA spanning bases 190–637 in the coding sequence was prepared by PCR using IMAGE clone 441025 as the template (purchased from American Type Culture Collection, Manassas, VA). The PCR product was gel-purified and ligated into the pCR4 plasmid. All subsequent steps were as described for mSEPP1.

### Statistical test

Means were compared using Student’s *t-*test.

## Results

**Figure 1:**
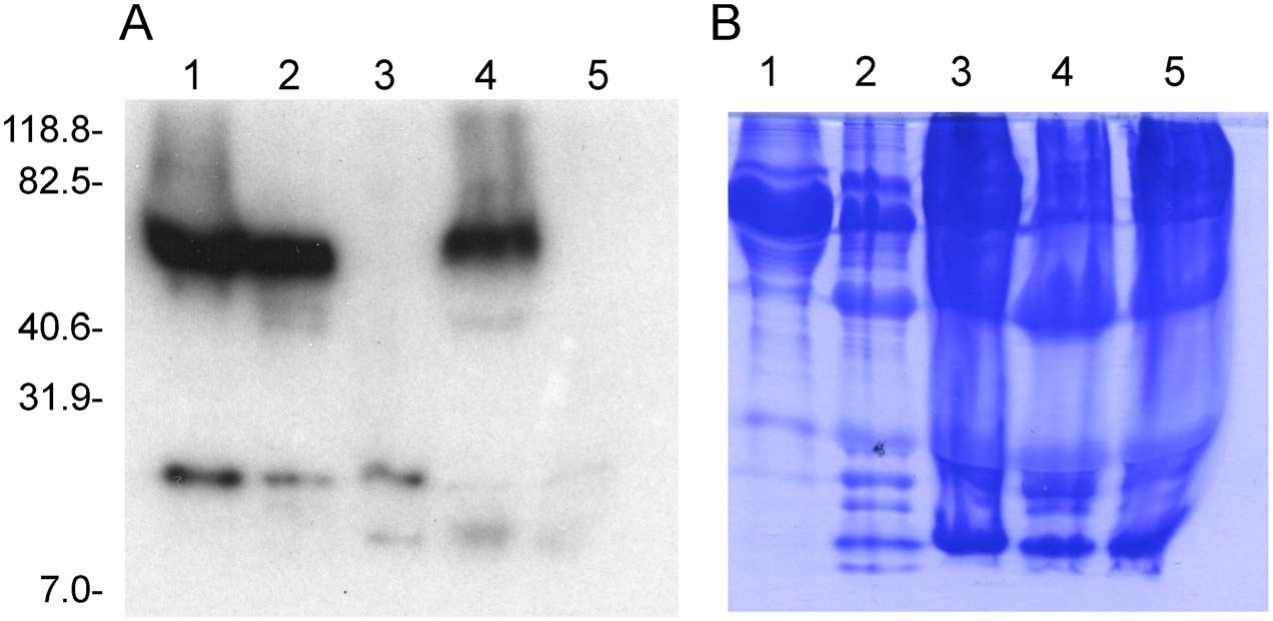
Selenium-containing proteins in milk whey detected by autoradiogram of an SDS-PAGE gel. Panel A is the autoradiogram of the gel and panel B depicts the gel stained by Coomassie blue. Dams that had been lactating 10–16 d were milked 24 h after administration of a tracer dose of ^75^SeO_3_^2−^ and the whey fraction of the milk was subjected to SDS-PAGE. ^75^Se-labeled plasma was included as a control. After drying, the gel was exposed to x-ray film. Lane 1 represents plasma (0.5 µl and 321 cpm applied) and lanes 2–5 represent whey (lane 2–1.5 µl and 260 cpm applied; lane 3–2 µl and 124 cpm applied; lane 4–1 µl and 288 cpm applied; lane 5–2 µl and 162 cpm applied). Deletion of selenoprotein genes in the dams is indicated in the lower half of the figure.

The identity of mouse milk proteins that contained selenium was sought. The milk was collected from dams 24 h after they had been injected with a tracer dose of ^75^SeO_3_^2−^ and whey from that milk was studied with SDS-PAGE autoradiography (Fig. 1A). Whey from C57BL/6 mice (lane 2) contained bands labeled with ^75^Se that corresponded to Sepp1 and Gpx3 in plasma (lane 1) as demonstrated in a previous publication [12]. No additional ^75^Se-containing bands were detected in lane 2. Study of whey from *Sepp1^−/−^* and *Gpx3^−/−^* dams (lanes 3 and 4, respectively) confirmed the identity of the ^75^Se-labeled bands in lane 2. Lane 5 was loaded with whey from a dam with deletion of both *Sepp1* and *Gpx3*. It contained no ^75^Se-labeled bands at the positions of Sepp1 or Gpx3. All lanes loaded with whey from dams with selenoprotein genes knocked out (lanes 3–5) contained a faint ^75^Se band at 13 kDa (Fig. 1A). Figure 1B shows that lanes 3 and 5 were loaded with more protein than the other lanes. More whey was loaded onto those lanes than onto lanes 2 and 4 so that enough ^75^Se would be present to detect labeled selenoproteins by autoradiography. These results confirm that Sepp1 and Gpx3, the 2 selenoproteins found in plasma, are also present in milk. The 13 kDa ^75^Se band might represent a selenium-containing protein that became detectable when ^75^Se availability increased as a consequence of deletion of *Sepp1* and/or *Gpx3*.

**Figure 2:**
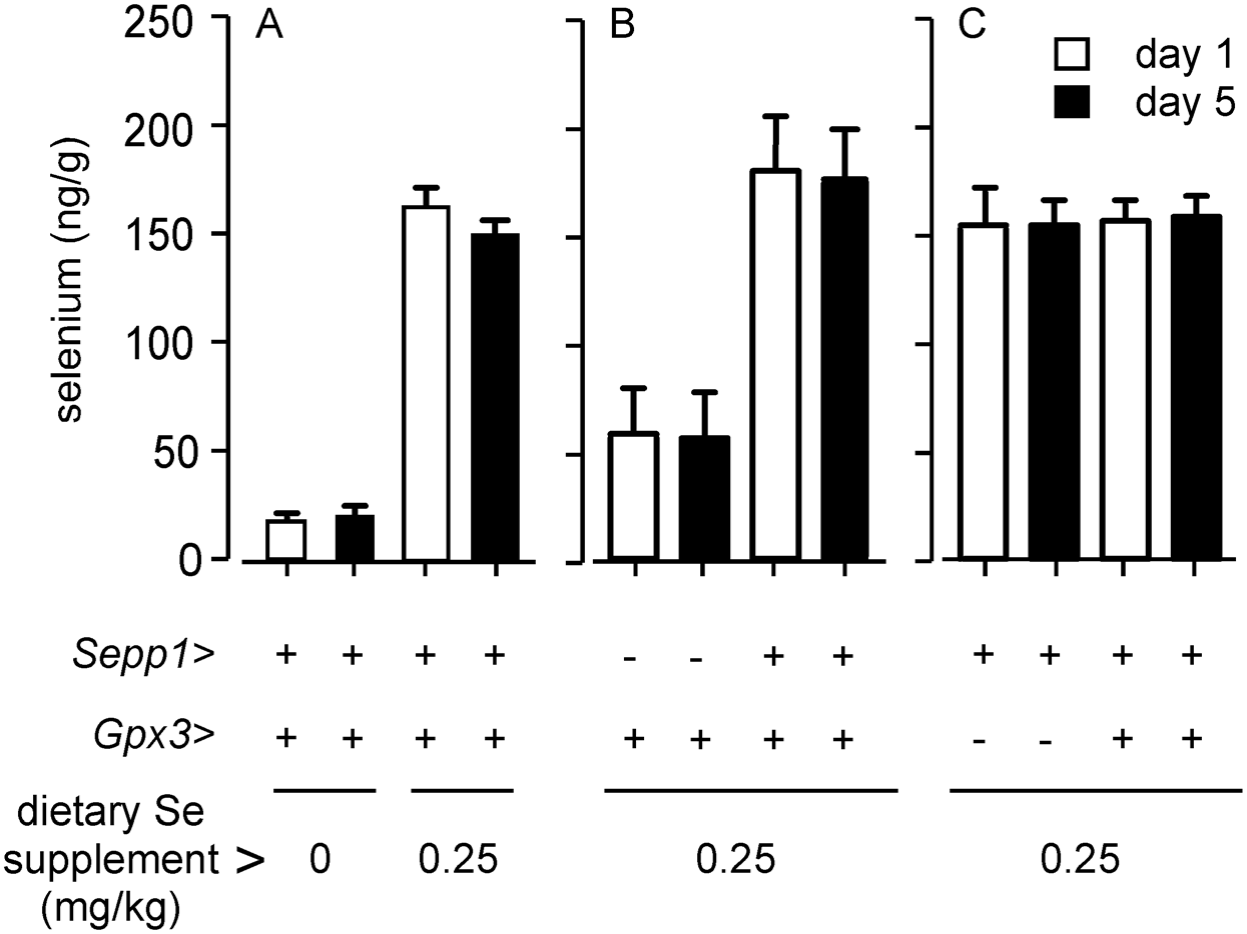
Effects of selenium intake and selenoprotein genotype of the dam on neonate selenium retention from milk. The experiments shown in each panel were carried out separately. Neonate whole-body selenium values depicted are means with S.D. indicated by a half bracket, n = 18–41. Dam genotypes and diet selenium supplements are shown in the lower half of the figure. Dams with gene deletions had been mated with C57BL/6 males, yielding neonates that were all heterozygous for the respective gene. Neonates were removed from the dam at day 1 or day 5 of life. They were weighed and euthanized and then assayed for selenium. The values in this figure were calculated from the raw data in Table S1. Weight gain and selenium gain over the 4 days are shown in Table 1.

During the week following birth, the mouse is totally dependent on mother’s milk for nourishment. We assessed the effect of maternal dietary selenium on selenium acquisition by neonates for the 4 days of life from day 1 to day 5. Figure 2A shows that neonates of selenium-deficient wild-type dams had much lower whole-body selenium concentrations (12%) than neonates of selenium-replete dams. The average amount of selenium retained by neonates from the milk of selenium-deficient dams over the 4 days was 18% of that retained by neonates of selenium-replete dams (Table 1). Thus, dietary selenium deficiency in the dam sharply reduces the selenium uptake and status of the neonate.

**Table 1:** Effect of dam genotype on nursing pup weight gain and selenium acquisition from day 1 to day 5 of life^a^.

dam	Neonatesstudied *n*		neonate bodyweight^b^ *g*		neonate seleniumcontent^b^ *ng*		weightgained^c^ *g*	seleniumacquired^c^ *ng*
	day 1	day 5	day 1	day 5	day 1	day 5		
selenium deficient	32	33	1.3±0.1	2.5±0.4	24±7	47±12	1.2	23
selenium replete	33	26	1.4±0.2	2.3±0.1	230±27	360±37	0.9	130
*Sepp1* ^−/−^	18	30	1.3±0.1	2.5±0.4	73±30	130±42	1.2	59
*Sepp1* ^+/+^	38	40	1.3±0.2	2.4±0.5	240±30	410±89	1.1	170
*Gpx3^−/^*	24	24	1.3±0.1	2.7±0.4	200±34	410±46	1.4	210
*Gpx3^+/^*	19	22	1.4±0.1	2.4±0.4	220±23	370±42	1.0	150

a See Table S1 for supporting data.

b Values are means ± 1 S.D.

c Values were calculated using day 1 and day 5 means.

The same protocol was used for experiments with *Sepp1^−/−^* and *Gpx3^−/−^* dams fed selenium-adequate diet. It was not possible to study selenium deficiency in *Sepp1^−/−^* dams because restricting their selenium intake would have caused severe brain injury and death [14]. Figure 2B shows that neonates of *Sepp1^−/−^* dams had 31% of the whole-body selenium concentration of neonates of *Sepp1^+/+^* dams, even though all dams had been fed diet supplemented with 0.25 mg selenium/kg. The average amount of selenium retained in the neonates of *Sepp1^−/−^* dams was 35% of that retained in the neonates of *Sepp1^+/+^* dams (Table 1). Deletion of *Gpx3* did not decrease neonate selenium concentration (Fig. 2C) or neonate selenium retention (Table 1). Thus, the *Sepp1^+/+^* genotype is important for selenium transfer from the selenium-replete dam to the neonate but the *Gpx3^+/+^* genotype is not.

**Figure 3:**
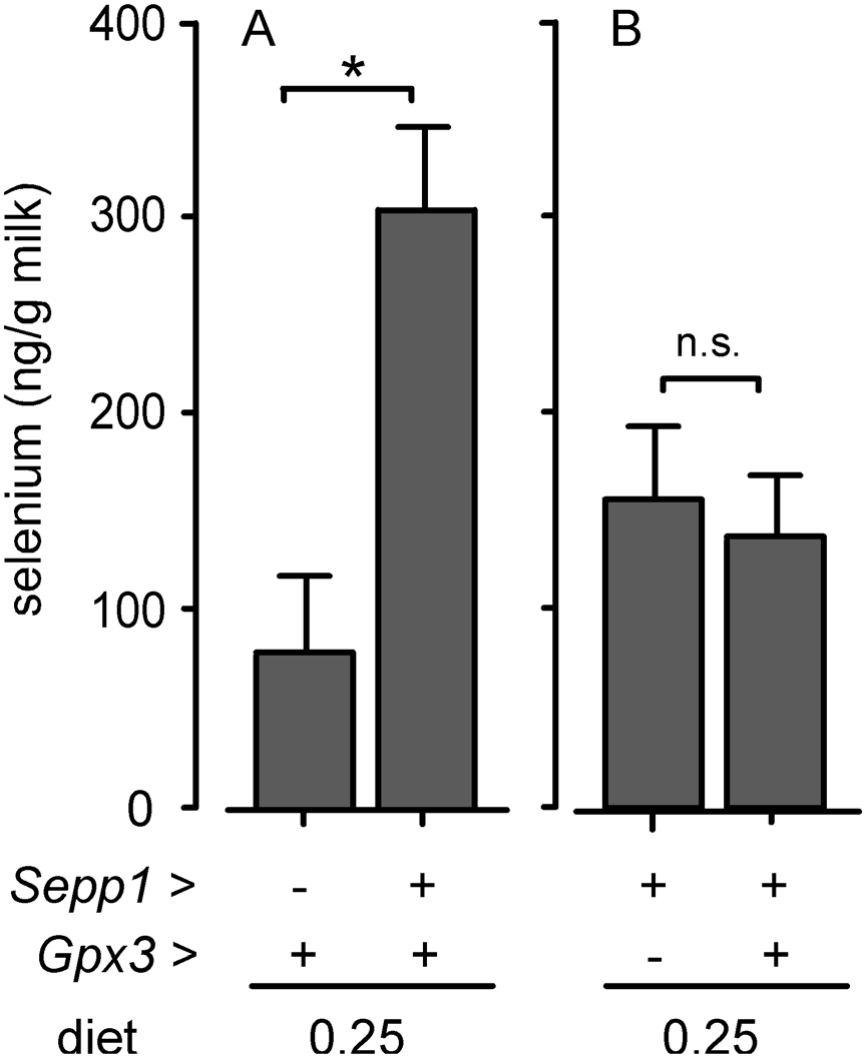
Effects of selenoprotein genotypes on milk selenium concentration. Panel A shows the effect of *Sepp1* deletion and panel B shows the effect of *Gpx3* deletion. Dam selenoprotein genotype is indicated below the graphs. All dams were fed diet supplemented with 0.25 mg selenium/kg. Values are means with 1 S.D. indicated by the half bracket, n = 4–9. The asterisk indicates that the two bars are significantly different by Student’s *t-*test, p<0.001. The n.s. designation indicates that the two bars are not significantly different by the same test. See Table S2 for supporting data.

The effect of selenoprotein gene deletion on the selenium content of milk was assessed. Efforts were made to control variations in milk selenium content by studying milk collected between the 10^th^ and 16^th^ days of lactation and by including temporal controls. Comparison of results from *Sepp1^−/−^* and *Sepp1^+/+^* dams indicates that 73% of milk selenium was associated with the *Sepp1^+/+^* genotype (Fig. 3A), while comparison of results from *Gpx3^−/−^* and *Gpx3^+/+^* dams indicates that an insignificant amount of milk selenium was associated with the *Gpx3^+/+^* genotype (Fig. 3B). Thus, the *Sepp1^+/+^* genotype accounts for much more of the selenium in milk from selenium-replete dams than does the *Gpx3^+/+^* genotype.

**Figure 4:**
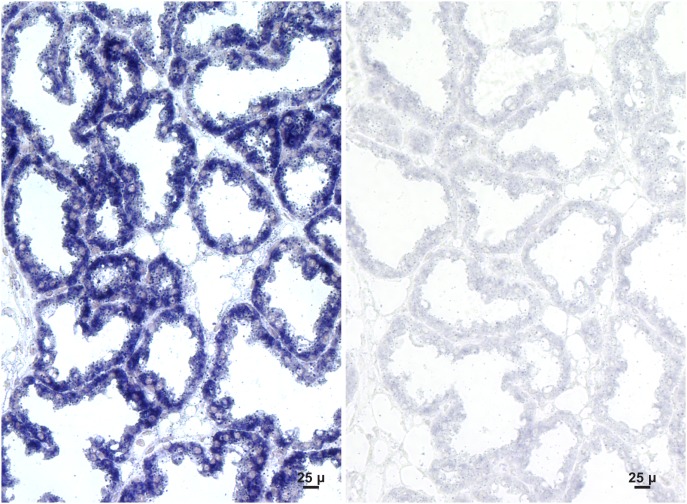
Sepp1 in situ hybridization of a lactating mouse mammary gland. Sepp1 mRNA is indicated by blue staining. Panel A was stained using the antisense Sepp1 riboprobe and panel B was stained using the sense Sepp1 riboprobe.

**Figure 5:**
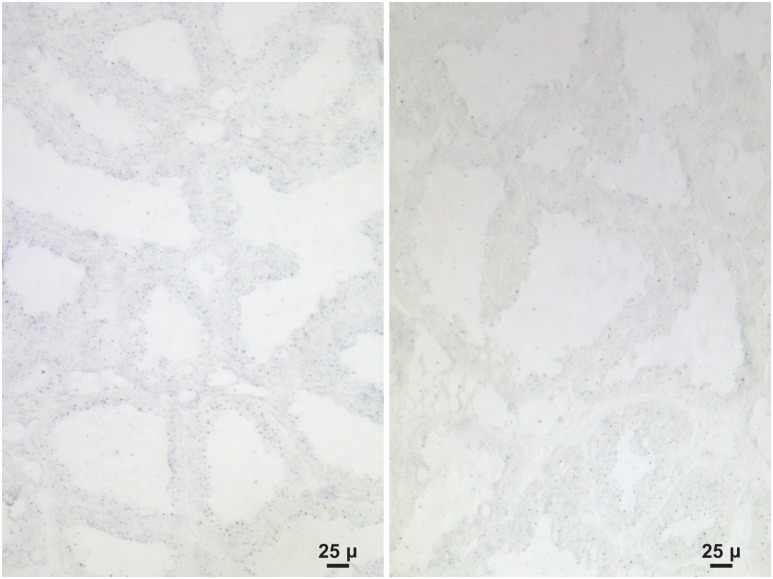
Gpx3 in situ hybridization of a lactating mouse mammary gland. Gpx3 mRNA was stained blue. Panel A was stained using the antisense Gpx3 riboprobe and panel B was stained using the sense Gpx3 riboprobe. See Figure S1 for a positive control.

Because *Sepp1* status is important in the transfer of selenium from the dam to the neonate and *Gpx3* status is not, in situ hybridization of the lactating mammary gland was carried out at day 13 of lactation. It showed that Sepp1 mRNA was present in the gland (Fig. 4) but that Gpx3 mRNA was not detected (Fig. 5, see also supplemental Fig. 1 for a positive control). This strongly suggests that milk Sepp1 is synthesized by the mammary gland but that little or no Gpx3 is synthesized by it. This finding leaves open the possibility that plasma is the source of milk Gpx3. It also raises the question of how the mammary gland acquires the selenium necessary for Sepp1 synthesis.

**Figure 6:**
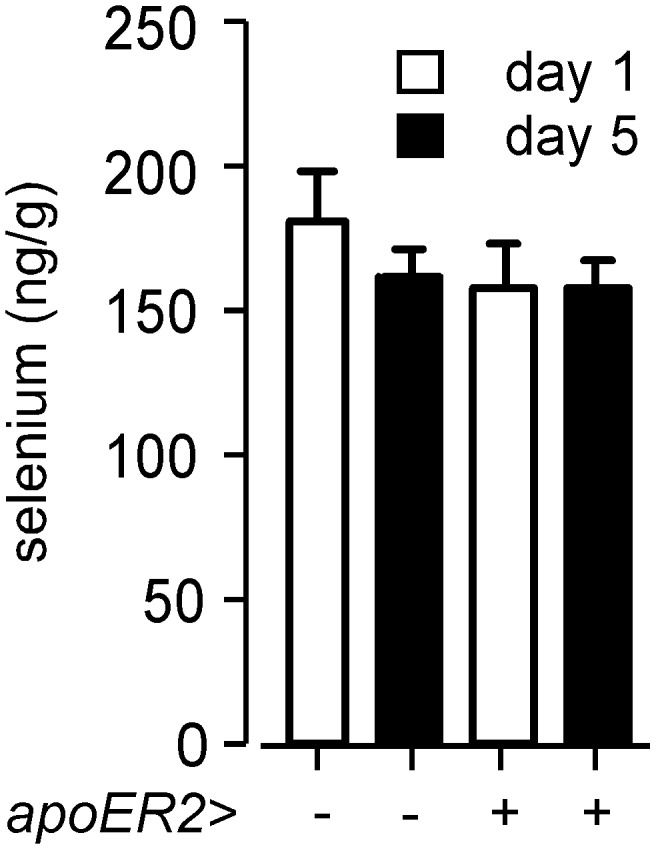
Effect of *apoER2* genotype of the dam on neonate selenium retention from milk. Neonate whole-body selenium values depicted are means with S.D. indicated by a half bracket, n = 28–42. Dam genotypes are shown in the lower half of the figure. All dams were fed diets supplemented with 0.25 mg selenium/kg. Dams with gene deletions had been mated with C57BL/6 males, yielding pups that were all heterozygous for the respective gene. Neonates were removed from the dam at day 1 or day 5 of life. They were weighed and euthanized before being assayed for selenium. See Table S3 for supporting data.

The testis exports a large amount of selenium in spermatozoa and acquires selenium to support this export by apoER2-mediated endocytosis of Sepp1 from the systemic circulation [17]. ApoER2 is the only receptor known to facilitate endocytosis of Sepp1 from the systemic circulation and male *apoER2^−/−^* mice are sterile. We sought a role for apoER2, a member of the low-density lipoprotein receptor family, in the transfer of selenium from the dam to the neonate. Deletion of maternal *apoER2* had no detectable effect on dam-to-neonate selenium transfer (Fig. 6). Thus, the mechanism of selenium uptake by the mammary gland does not appear to involve apoER2-mediated endocytosis of Sepp1 from maternal plasma. The mechanism by which the mammary gland is supplied with selenium remains unknown.

## Discussion

The experiments reported here took advantage of gene deletion techniques to assess the effectiveness of Sepp1 and Gpx3 in transferring selenium from the selenium-replete dam to its nursing neonate. Deletion of *Sepp1* reduced selenium transfer to the neonate by 65% while no decreased transfer was detected when *Gpx3* was deleted (Fig. 2 & Table 1). Supporting the transfer experiments, analysis of milk revealed that the *Sepp1^+/+^* genotype accounted for 73% of the selenium in milk while the amount accounted for by the *Gpx3^+/+^* genotype was not statistically significant. Further support for the predominance of Sepp1 over Gpx3 in the transport of selenium by milk was the demonstration that the lactating mammary gland expressed Sepp1 mRNA (Fig. 4) but that Gpx3 mRNA was not detectable in it by in situ hybridization (Fig. 5). Thus, the *Sepp1^+/+^* genotype is important for selenium transfer from the selenium-replete dam to the neonate but the *Gpx3^+/+^* genotype is not.

It is likely that the maternal *Sepp1^−/−^* genotype decreases selenium transfer from dam to nursing pup through two mechanisms. One of the mechanisms is the elimination of selenium-rich Sepp1 from milk as demonstrated in figure 1. The other mechanism is decreasing the whole-body selenium content of the dam [9], [11], thereby reducing the amount of maternal selenium available for incorporation into milk. *Sepp1* deletion reduces whole-body selenium of non-pregnant female mice by only 21% [11], suggesting that the 73% reduction of milk selenium by *Sepp1* deletion is mainly attributable to the absence of Sepp1 from the milk. However, the relative contributions of these mechanisms to the decrease in milk selenium by the *Sepp1^−/−^* genotype were not determined directly.

Previous work made clear that milk selenium is present in several forms. As confirmed here, the selenoproteins Sepp1 and Gpx3 are among those forms. A large amount of milk selenium–perhaps 30% of it–is present in small-molecule form that has not been characterized [3]. The large fraction of milk selenium in small-molecule form contrasts with the much smaller fraction (less than 3%) of plasma selenium in small-molecule form [12]. The small-molecule forms in plasma include excretory metabolites produced by the liver–largely selenosugars and other methylated compounds [18], [19]. These excretory forms seem unlikely to account for much of the small-molecule selenium in milk because they are cleared from the blood by the kidneys and appear in the urine.

Little is known about small-molecule transport forms of selenium. At least one small-molecule form in plasma transports selenium to tissues because mice with both *Sepp1* and *Gpx3* deleted are viable when fed high-selenium diet [12]. This small-molecule transport form in plasma has not yet been characterized and further work is needed to characterize it and the small-molecule selenium in milk.

When present in the diet of lactating individuals, selenomethionine contributes to the selenium in milk. Feeding selenium as selenomethionine to the lactating rat dam results in a higher milk selenium concentration than does feeding selenite [5]. Gel filtration of defatted and dialyzed human milk was shown to have 9 selenium peaks with apparent molecular weights of 8 to 150 kDa [3]. The likely reason that more selenium-containing proteins were detected in human milk than in mouse milk is that humans ingest selenomethionine as their major dietary form of selenium and our mice were fed selenium only as selenite. Selenomethionine is present at methionine positions in many proteins, but selenite, when fed at physiological levels of selenium, does not enter proteins nonspecifically and provides selenium only for selenoproteins. Thus, milk from free-living animals and humans can be expected to contain many proteins that contain selenium in selenomethionine form.

Both Sepp1 and Gpx3 are enzymes with peroxidase activity [20]. It is possible that they have enzymatic functions in milk. They might protect against peroxides in the milk or in the gastrointestinal tract of the neonate. Such a function seems likely for Gpx3 because no evidence that it has a selenium transfer function was found in these studies of selenium-replete mice.

In conclusion, the *Sepp1^+/+^* genotype is responsible for most of the selenium transfer from the dam to the neonate under selenium-adequate conditions. Sepp1 is synthesized in the mammary gland and the *Sepp1* genotype accounts for most of the selenium in mouse milk. The Gpx3 present in milk accounts for much less selenium than does Sepp1 and, thus, Gpx3 is responsible for very little of the selenium transferred from the dam to the neonate under selenium-adequate conditions. Unidentified small-molecule forms of selenium are present in milk and contribute to the transfer of selenium from the dam to the neonate.

In free-living animals the effect of proteins containing selenomethionine will be superimposed on the effects of Sepp1, Gpx3, and the small-molecule forms of selenium. However, selenoproteins can be expected to maintain their selenium better than selenomethionine-containing proteins under selenium-deficient conditions. Thus, we speculate that Sepp1, and possibly Gpx3, will be of great importance in transferring maternal selenium to the nursing neonate when dietary selenium is limiting in humans and free-living animals.

## Supporting Information

Figure S1 **This figure shows a tissue, wild-type mouse kidney cortex, that expresses Gpx3 mRNA (stained).** It serves as a positive control for figure 5 in the manuscript, which does not detect Gpx3 mRNA in the lactating mammary gland.
(TIF)

Table S1 **Supporting data for** **Table 1** **and** **Figure 2** **.**
(DOCX)

Table S2 **Supporting data for** **Figure 3** **.**
(DOCX)

Table S3 **Supporting data for** **Figure 6** **.**
(DOCX)
