# Supplementary figures and images for: Selenoprotein P Is the Major Selenium Transport Protein in Mouse Milk
Source: PLoS One. 2014 Jul 28;9(7):e103486. doi: 10.1371/journal.pone.0103486 (PMC4113432; doi:10.1371/journal.pone.0103486)

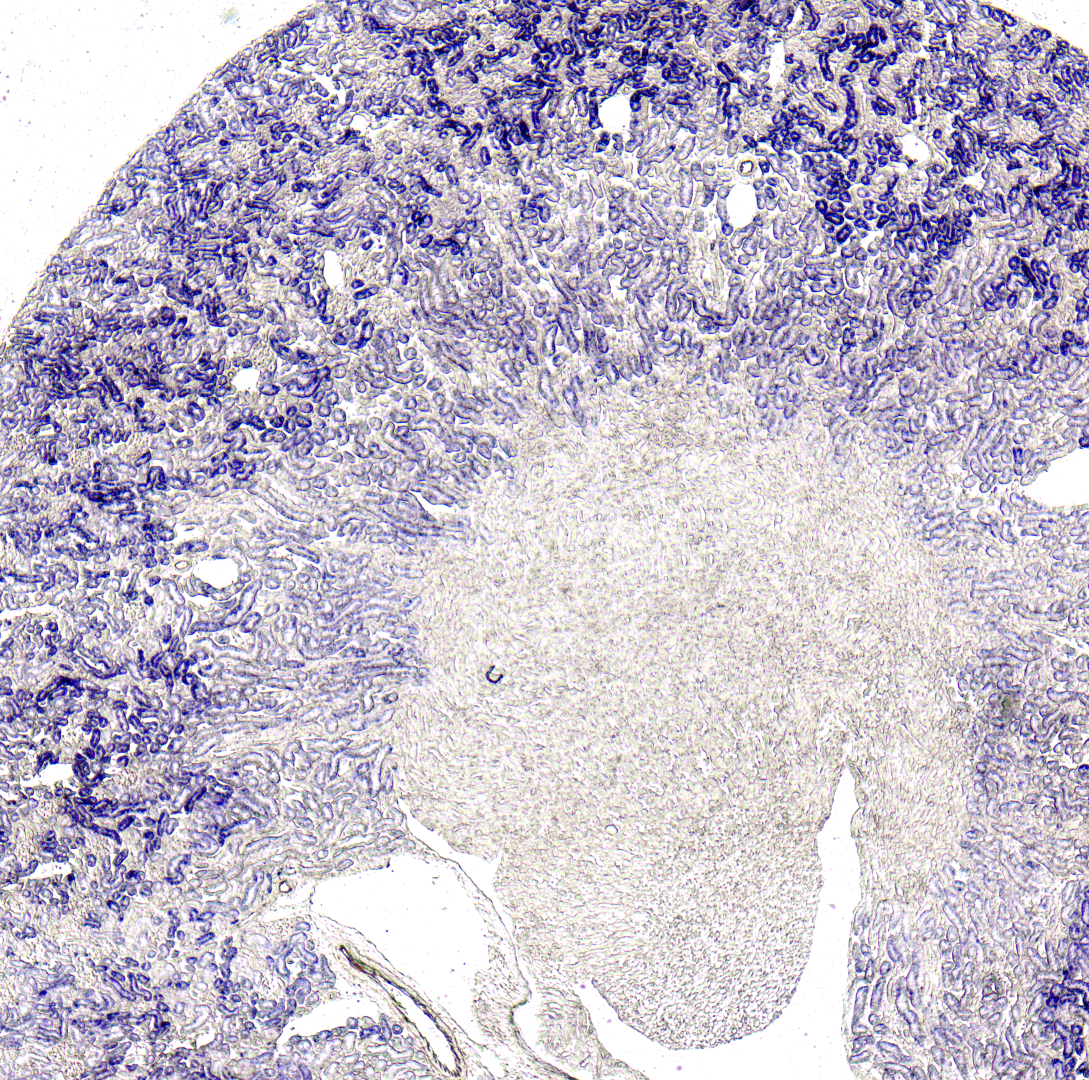

Supplement: Figure S1 — This figure shows a tissue, wild-type mouse kidney cortex, that expresses Gpx3 mRNA (stained). It serves as a positive control for figure 5 in the manuscript, which does not detect Gpx3 mRNA in the lactating mammary gland. (TIF) [file pone.0103486.s001.tif]
