# Supplementary material for: Selenoprotein P Is the Major Selenium Transport Protein in Mouse Milk
Source: PLoS One. 2014 Jul 28;9(7):e103486. doi: 10.1371/journal.pone.0103486 (PMC4113432; doi:10.1371/journal.pone.0103486)
Supplement: Table S1 — Supporting data for Table 1 and Figure 2 . (DOCX) [file pone.0103486.s002.docx]

Table S1 (supporting data for Table 1 and Figure 2)

| dam ID# | diet | neonate ID# | dam strain | neonate genotype | age (day) | weight (g) | whole body Se | |
| --- | --- | --- | --- | --- | --- | --- | --- | --- |
|  |  |  |  |  |  |  | (ng) | (ng/g) |
| 10.203 | 0.25 ppm Se | p1 | Sepp1-/- | ht | 1 | 1.380 | 85 | 84.6 |
|  | 0.25 ppm Se | p2 | Sepp1-/- | ht | 1 | 1.334 | 104 | 104.3 |
|  | 0.25 ppm Se | p3 | Sepp1-/- | ht | 1 | 1.376 | 78 | 78.0 |
|  | 0.25 ppm Se | p4 | Sepp1-/- | ht | 1 | 1.393 | 161 | 160.6 |
|  | 0.25 ppm Se | p5 | Sepp1-/- | ht | 1 | 1.358 | 110 | 109.6 |
| 10.220 | 0.25 ppm Se | p1 | Sepp1-/- | ht | 1 | 1.262 | 72 | 71.8 |
|  | 0.25 ppm Se | p2 | Sepp1-/- | ht | 1 | 1.379 | 62 | 61.8 |
|  | 0.25 ppm Se | p3 | Sepp1-/- | ht | 1 | 1.438 | 53 | 52.8 |
|  | 0.25 ppm Se | p4 | Sepp1-/- | ht | 1 | 1.375 | 59 | 58.9 |
|  | 0.25 ppm Se | p5 | Sepp1-/- | ht | 1 | 1.245 | 38 | 38.2 |
|  | 0.25 ppm Se | p6 | Sepp1-/- | ht | 1 | 1.292 | 34 | 33.7 |
|  | 0.25 ppm Se | p7 | Sepp1-/- | ht | 1 | 1.341 | 53 | 52.6 |
|  | 0.25 ppm Se | p8 | Sepp1-/- | ht | 1 | 1.315 | 55 | 54.7 |
| 10.221 | 0.25 ppm Se | p2 | Sepp1-/- | ht | 1 | 1.161 | 87 | 87.1 |
|  | 0.25 ppm Se | p3 | Sepp1-/- | ht | 1 | 1.321 | 58 | 58.4 |
|  | 0.25 ppm Se | p4 | Sepp1-/- | ht | 1 | 1.009 | 67 | 66.9 |
|  | 0.25 ppm Se | p5 | Sepp1-/- | ht | 1 | 1.210 | 76 | 75.7 |
|  | 0.25 ppm Se | p6 | Sepp1-/- | ht | 1 | 1.210 | 66 | 65.7 |
| 10.203 | 0.25 ppm Se | p2 | Sepp1-/- | ht | 5 | 2.109 | 93 | 92.6 |
|  | 0.25 ppm Se | p3 | Sepp1-/- | ht | 5 | 2.687 | 94 | 94.3 |
|  | 0.25 ppm Se | p4 | Sepp1-/- | ht | 5 | 2.371 | 186 | 185.9 |
|  | 0.25 ppm Se | p5 | Sepp1-/- | ht | 5 | 1.797 | 87 | 87.0 |
|  | 0.25 ppm Se | p6 | Sepp1-/- | ht | 5 | 2.647 | 179 | 179.5 |
|  | 0.25 ppm Se | p7 | Sepp1-/- | ht | 5 | 2.593 | 144 | 144.4 |
| 10.221 | 0.25 ppm Se | p1 | Sepp1-/- | ht | 5 | 1.526 | 75 | 75.2 |
|  | 0.25 ppm Se | p2 | Sepp1-/- | ht | 5 | 2.368 | 98 | 98.3 |
|  | 0.25 ppm Se | p3 | Sepp1-/- | ht | 5 | 2.951 | 106 | 105.6 |
|  | 0.25 ppm Se | p4 | Sepp1-/- | ht | 5 | 2.308 | 94 | 93.9 |
|  | 0.25 ppm Se | p5 | Sepp1-/- | ht | 5 | 2.902 | 104 | 104.5 |
|  | 0.25 ppm Se | p6 | Sepp1-/- | ht | 5 | 2.660 | 83 | 83.0 |
| 10.221 | 0.25 ppm Se | p1 | Sepp1-/- | ht | 5 | 2.955 | 112 | 112.3 |
|  | 0.25 ppm Se | p2 | Sepp1-/- | ht | 5 | 3.104 | 199 | 199.0 |
|  | 0.25 ppm Se | p3 | Sepp1-/- | ht | 5 | 3.227 | 137 | 136.5 |
|  | 0.25 ppm Se | p4 | Sepp1-/- | ht | 5 | 3.003 | 143 | 143.2 |
|  | 0.25 ppm Se | p5 | Sepp1-/- | ht | 5 | 3.129 | 151 | 151.4 |
| 10.281 | 0.25 ppm Se | p1 | Sepp1-/- | ht | 5 | 2.429 | 157 | 156.9 |
|  | 0.25 ppm Se | p2 | Sepp1-/- | ht | 5 | 2.342 | 80 | 79.9 |
|  | 0.25 ppm Se | p3 | Sepp1-/- | ht | 5 | 1.770 | 183 | 183.2 |
|  | 0.25 ppm Se | p4 | Sepp1-/- | ht | 5 | 2.551 | 139 | 139.0 |
|  | 0.25 ppm Se | p5 | Sepp1-/- | ht | 5 | 2.245 | 105 | 105.1 |
|  | 0.25 ppm Se | p6 | Sepp1-/- | ht | 5 | 2.248 | 124 | 123.9 |
| 10.281 | 0.25 ppm Se | p2 | Sepp1-/- | ht | 5 | 2.871 | 222 | 221.9 |
|  | 0.25 ppm Se | p3 | Sepp1-/- | ht | 5 | 2.217 | 96 | 95.6 |
|  | 0.25 ppm Se | p4 | Sepp1-/- | ht | 5 | 2.372 | 97 | 97.5 |
|  | 0.25 ppm Se | p5 | Sepp1-/- | ht | 5 | 2.451 | 148 | 147.6 |
|  | 0.25 ppm Se | p6 | Sepp1-/- | ht | 5 | 1.843 | 145 | 144.7 |
|  | 0.25 ppm Se | p7 | Sepp1-/- | ht | 5 | 1.903 | 209 | 208.8 |
|  | 0.25 ppm Se | p8 | Sepp1-/- | ht | 5 | 2.752 | 176 | 175.9 |
| 10.226 | 0.25 ppm Se | p1 | Sepp1+/+ | ht | 1 | 1.070 | 237 | 236.7 |
|  | 0.25 ppm Se | p2 | Sepp1+/+ | wt | 1 | 1.039 | 208 | 208.2 |
|  | 0.25 ppm Se | p3 | Sepp1+/+ | wt | 1 | 1.163 | 227 | 227.4 |
|  | 0.25 ppm Se | p4 | Sepp1+/+ | ht | 1 | 1.041 | 227 | 226.6 |
|  | 0.25 ppm Se | p5 | Sepp1+/+ | wt | 1 | 1.041 | 223 | 222.7 |
|  | 0.25 ppm Se | p6 | Sepp1+/+ | wt | 1 | 1.086 | 228 | 228.1 |
| 10.227 | 0.25 ppm Se | p1 | Sepp1+/+ | ht | 1 | 1.200 | 234 | 233.9 |
|  | 0.25 ppm Se | p2 | Sepp1+/+ | wt | 1 | 1.498 | 258 | 257.8 |
|  | 0.25 ppm Se | p3 | Sepp1+/+ | wt | 1 | 1.386 | 279 | 279.0 |
|  | 0.25 ppm Se | p4 | Sepp1+/+ | wt | 1 | 1.281 | 287 | 287.5 |
|  | 0.25 ppm Se | p5 | Sepp1+/+ | ht | 1 | 1.338 | 245 | 245.0 |
|  | 0.25 ppm Se | p7 | Sepp1+/+ | wt | 1 | 1.462 | 237 | 236.6 |
|  | 0.25 ppm Se | p8 | Sepp1+/+ | ht | 1 | 1.279 | 232 | 232.1 |
| 10.228 | 0.25 ppm Se | p1 | Sepp1+/+ | wt | 1 | 1.399 | 235 | 234.6 |
|  | 0.25 ppm Se | p2 | Sepp1+/+ | ht | 1 | 1.204 | 197 | 197.0 |
|  | 0.25 ppm Se | p3 | Sepp1+/+ | wt | 1 | 1.433 | 228 | 228.3 |
|  | 0.25 ppm Se | p4 | Sepp1+/+ | wt | 1 | 1.390 | 232 | 231.9 |
|  | 0.25 ppm Se | p5 | Sepp1+/+ | ht | 1 | 1.401 | 215 | 214.6 |
|  | 0.25 ppm Se | p6 | Sepp1+/+ | ht | 1 | 1.391 | 254 | 253.9 |
|  | 0.25 ppm Se | p7 | Sepp1+/+ | wt | 1 | 1.529 | 313 | 313.1 |
|  | 0.25 ppm Se | p8 | Sepp1+/+ | ht | 1 | 1.502 | 339 | 338.6 |
| 10.230 | 0.25 ppm Se | p3 | Sepp1+/+ | wt | 1 | 1.449 | 214 | 213.6 |
|  | 0.25 ppm Se | p4 | Sepp1+/+ | wt | 1 | 1.319 | 220 | 220.0 |
|  | 0.25 ppm Se | p5 | Sepp1+/+ | wt | 1 | 1.457 | 253 | 253.2 |
|  | 0.25 ppm Se | p6 | Sepp1+/+ | wt | 1 | 1.302 | 221 | 220.6 |
|  | 0.25 ppm Se | p7 | Sepp1+/+ | ht | 1 | 1.235 | 203 | 202.8 |
| 10.230 | 0.25 ppm Se | p2 | Sepp1+/+ | ht | 1 | 1.702 | 257 | 256.7 |
|  | 0.25 ppm Se | p3 | Sepp1+/+ | wt | 1 | 1.450 | 222 | 222.0 |
|  | 0.25 ppm Se | p4 | Sepp1+/+ | ht | 1 | 1.595 | 228 | 228.2 |
|  | 0.25 ppm Se | p5 | Sepp1+/+ | ht | 1 | 1.684 | 285 | 284.9 |
|  | 0.25 ppm Se | p6 | Sepp1+/+ | wt | 1 | 1.571 | 238 | 238.2 |
| 10.286 | 0.25 ppm Se | p1 | Sepp1+/+ | ht | 1 | 1.115 | 219 | 218.9 |
|  | 0.25 ppm Se | p2 | Sepp1+/+ | ht | 1 | 1.220 | 216 | 215.9 |
|  | 0.25 ppm Se | p3 | Sepp1+/+ | wt | 1 | 1.237 | 238 | 238.0 |
|  | 0.25 ppm Se | p4 | Sepp1+/+ | wt | 1 | 1.327 | 260 | 260.0 |
|  | 0.25 ppm Se | p5 | Sepp1+/+ | ht | 1 | 1.323 | 269 | 269.5 |
| 10.226 | 0.25 ppm Se | p2 | Sepp1+/+ | wt | 5 | 1.476 | 249 | 248.9 |
|  | 0.25 ppm Se | p3 | Sepp1+/+ | ht | 5 | 1.701 | 267 | 267.1 |
|  | 0.25 ppm Se | p4 | Sepp1+/+ | ht | 5 | 1.790 | 255 | 255.4 |
|  | 0.25 ppm Se | p5 | Sepp1+/+ | ht | 5 | 1.912 | 277 | 277.0 |
| 10.227 | 0.25 ppm Se | p1 | Sepp1+/+ | wt | 5 | 2.312 | 493 | 492.7 |
|  | 0.25 ppm Se | p2 | Sepp1+/+ | ht | 5 | 2.499 | 466 | 466.1 |
|  | 0.25 ppm Se | p3 | Sepp1+/+ | wt | 5 | 2.522 | 557 | 557.1 |
|  | 0.25 ppm Se | p4 | Sepp1+/+ | wt | 5 | 2.332 | 413 | 413.5 |
|  | 0.25 ppm Se | p5 | Sepp1+/+ | wt | 5 | 2.379 | 467 | 467.5 |
|  | 0.25 ppm Se | p6 | Sepp1+/+ | wt | 5 | 2.290 | 430 | 430.3 |
| 10.228 | 0.25 ppm Se | p1 | Sepp1+/+ | wt | 5 | 2.225 | 387 | 387.4 |
|  | 0.25 ppm Se | p2 | Sepp1+/+ | wt | 5 | 2.075 | 295 | 295.3 |
|  | 0.25 ppm Se | p3 | Sepp1+/+ | ht | 5 | 2.216 | 391 | 391.1 |
|  | 0.25 ppm Se | p4 | Sepp1+/+ | ht | 5 | 2.328 | 380 | 379.9 |
|  | 0.25 ppm Se | p5 | Sepp1+/+ | wt | 5 | 1.987 | 402 | 402.4 |
|  | 0.25 ppm Se | p6 | Sepp1+/+ | wt | 5 | 2.195 | 414 | 414.0 |
|  | 0.25 ppm Se | p7 | Sepp1+/+ | wt | 5 | 2.160 | 415 | 415.2 |
|  | 0.25 ppm Se | p8 | Sepp1+/+ | ht | 5 | 2.469 | 393 | 392.6 |
|  | 0.25 ppm Se | p9 | Sepp1+/+ | wt | 5 | 1.200 | 245 | 244.9 |
| 10.230 | 0.25 ppm Se | p1 | Sepp1+/+ | ht | 5 | 2.430 | 419 | 419.2 |
|  | 0.25 ppm Se | p2 | Sepp1+/+ | ht | 5 | 2.570 | 445 | 445.4 |
|  | 0.25 ppm Se | p3 | Sepp1+/+ | ht | 5 | 2.547 | 405 | 404.7 |
|  | 0.25 ppm Se | p4 | Sepp1+/+ | ht | 5 | 2.461 | 380 | 380.5 |
|  | 0.25 ppm Se | p5 | Sepp1+/+ | ht | 5 | 2.654 | 406 | 406.3 |
|  | 0.25 ppm Se | p6 | Sepp1+/+ | wt | 5 | 1.733 | 310 | 309.5 |
|  | 0.25 ppm Se | p7 | Sepp1+/+ | wt | 5 | 1.459 | 282 | 282.5 |
| 10.284 | 0.25 ppm Se | p1 | Sepp1+/+ | wt | 5 | 2.353 | 413 | 413.0 |
|  | 0.25 ppm Se | p2 | Sepp1+/+ | wt | 5 | 2.628 | 456 | 456.5 |
|  | 0.25 ppm Se | p3 | Sepp1+/+ | ht | 5 | 2.227 | 385 | 384.6 |
|  | 0.25 ppm Se | p4 | Sepp1+/+ | wt | 5 | 2.359 | 444 | 444.0 |
|  | 0.25 ppm Se | p5 | Sepp1+/+ | wt | 5 | 2.066 | 407 | 406.8 |
| 10.285 | 0.25 ppm Se | p1 | Sepp1+/+ | ht | 5 | 3.236 | 448 | 447.9 |
|  | 0.25 ppm Se | p2 | Sepp1+/+ | ht | 5 | 3.479 | 485 | 485.0 |
|  | 0.25 ppm Se | p3 | Sepp1+/+ | ht | 5 | 3.419 | 465 | 464.6 |
|  | 0.25 ppm Se | p4 | Sepp1+/+ | ht | 5 | 2.812 | 373 | 373.2 |
| 10.295 | 0.25 ppm Se | p1 | Sepp1+/+ | ht | 5 | 2.775 | 455 | 454.8 |
|  | 0.25 ppm Se | p2 | Sepp1+/+ | wt | 5 | 2.665 | 453 | 453.3 |
|  | 0.25 ppm Se | p3 | Sepp1+/+ | ht | 5 | 2.620 | 499 | 498.6 |
|  | 0.25 ppm Se | p4 | Sepp1+/+ | wt | 5 | 2.852 | 575 | 575.2 |
|  | 0.25 ppm Se | p5 | Sepp1+/+ | ht | 5 | 2.784 | 650 | 649.8 |
| G3.7-3 | 0.25 ppm Se | p1 | Gpx3-/- | hm | 1 | 1.549 | 239 | 154.0 |
|  | 0.25 ppm Se | p2 | Gpx3-/- | hm | 1 | 1.354 | 199 | 147.2 |
|  | 0.25 ppm Se | p3 | Gpx3-/- | hm | 1 | 1.298 | 174 | 134.1 |
|  | 0.25 ppm Se | p4 | Gpx3-/- | hm | 1 | 1.261 | 186 | 147.2 |
|  | 0.25 ppm Se | p5 | Gpx3-/- | hm | 1 | 1.045 | 136 | 130.4 |
|  | 0.25 ppm Se | p6 | Gpx3-/- | hm | 1 | 1.451 | 208 | 143.7 |
|  | 0.25 ppm Se | p7 | Gpx3-/- | hm | 1 | 1.244 | 187 | 150.4 |
|  | 0.25 ppm Se | p8 | Gpx3-/- | hm | 1 | 1.392 | 177 | 127.2 |
|  | 0.25 ppm Se | p9 | Gpx3-/- | hm | 1 | 1.427 | 202 | 141.8 |
| G3.15-2 | 0.25 ppm Se | p1 | Gpx3-/- | hm | 1 | 1.372 | 202 | 147.2 |
|  | 0.25 ppm Se | p2 | Gpx3-/- | hm | 1 | 1.196 | 184 | 153.7 |
|  | 0.25 ppm Se | p3 | Gpx3-/- | hm | 1 | 1.405 | 225 | 160.0 |
|  | 0.25 ppm Se | p4 | Gpx3-/- | hm | 1 | 1.315 | 183 | 139.0 |
|  | 0.25 ppm Se | p5 | Gpx3-/- | hm | 1 | 1.317 | 188 | 142.9 |
|  | 0.25 ppm Se | p6 | Gpx3-/- | hm | 1 | 1.152 | 170 | 147.8 |
|  | 0.25 ppm Se | p7 | Gpx3-/- | hm | 1 | 1.326 | 197 | 148.9 |
|  | 0.25 ppm Se | p8 | Gpx3-/- | hm | 1 | 1.556 | 217 | 139.4 |
|  | 0.25 ppm Se | p9 | Gpx3-/- | hm | 1 | 0.995 | 153 | 153.4 |
| G3.7-1 | 0.25 ppm Se | p1 | Gpx3-/- | hm | 1 | 1.398 | 230 | 164.3 |
|  | 0.25 ppm Se | p2 | Gpx3-/- | hm | 1 | 1.357 | 239 | 176.3 |
|  | 0.25 ppm Se | p3 | Gpx3-/- | hm | 1 | 1.397 | 254 | 181.5 |
|  | 0.25 ppm Se | p4 | Gpx3-/- | hm | 1 | 1.463 | 289 | 197.7 |
|  | 0.25 ppm Se | p5 | Gpx3-/- | hm | 1 | 1.193 | 195 | 163.4 |
|  | 0.25 ppm Se | p6 | Gpx3-/- | hm | 1 | 1.284 | 232 | 180.7 |
| G3.6-2 | 0.25 ppm Se | p1 | Gpx3-/- | hm | 5 | 2.800 | 410 | 146.4 |
|  | 0.25 ppm Se | p2 | Gpx3-/- | hm | 5 | 2.957 | 424 | 143.4 |
|  | 0.25 ppm Se | p3 | Gpx3-/- | hm | 5 | 2.902 | 414 | 142.5 |
|  | 0.25 ppm Se | p4 | Gpx3-/- | hm | 5 | 3.011 | 435 | 144.5 |
|  | 0.25 ppm Se | p5 | Gpx3-/- | hm | 5 | 3.144 | 447 | 142.1 |
|  | 0.25 ppm Se | p6 | Gpx3-/- | hm | 5 | 2.829 | 396 | 140.0 |
|  | 0.25 ppm Se | p7 | Gpx3-/- | hm | 5 | 2.566 | 366 | 142.8 |
|  | 0.25 ppm Se | p8 | Gpx3-/- | hm | 5 | 2.979 | 424 | 142.3 |
| G3.4-3 | 0.25 ppm Se | p1 | Gpx3-/- | hm | 5 | 3.010 | 460 | 152.8 |
|  | 0.25 ppm Se | p2 | Gpx3-/- | hm | 5 | 2.160 | 352 | 163.1 |
|  | 0.25 ppm Se | p3 | Gpx3-/- | hm | 5 | 2.880 | 473 | 164.1 |
|  | 0.25 ppm Se | p4 | Gpx3-/- | hm | 5 | 3.050 | 486 | 159.2 |
|  | 0.25 ppm Se | p5 | Gpx3-/- | hm | 5 | 2.960 | 448 | 151.2 |
|  | 0.25 ppm Se | p6 | Gpx3-/- | hm | 5 | 2.900 | 483 | 166.4 |
|  | 0.25 ppm Se | p7 | Gpx3-/- | hm | 5 | 3.040 | 425 | 139.8 |
|  | 0.25 ppm Se | p8 | Gpx3-/- | hm | 5 | 2.880 | 475 | 164.9 |
| G3.5-1 | 0.25 ppm Se | p1 | Gpx3-/- | hm | 5 | 2.290 | 372 | 162.3 |
|  | 0.25 ppm Se | p2 | Gpx3-/- | hm | 5 | 2.170 | 358 | 165.1 |
|  | 0.25 ppm Se | p3 | Gpx3-/- | hm | 5 | 2.300 | 398 | 173.0 |
|  | 0.25 ppm Se | p4 | Gpx3-/- | hm | 5 | 2.240 | 339 | 151.5 |
|  | 0.25 ppm Se | p5 | Gpx3-/- | hm | 5 | 2.160 | 351 | 162.3 |
|  | 0.25 ppm Se | p6 | Gpx3-/- | hm | 5 | 2.270 | 379 | 167.1 |
|  | 0.25 ppm Se | p7 | Gpx3-/- | hm | 5 | 2.300 | 390 | 169.4 |
|  | 0.25 ppm Se | p8 | Gpx3-/- | hm | 5 | 2.250 | 352 | 156.7 |
| G3.9-1 | 0.25 ppm Se | p1 | Gpx3+/+ | wt | 1 | 1.528 | 247 | 161.9 |
|  | 0.25 ppm Se | p2 | Gpx3+/+ | wt | 1 | 1.321 | 194 | 147.0 |
|  | 0.25 ppm Se | p3 | Gpx3+/+ | wt | 1 | 1.547 | 240 | 155.4 |
|  | 0.25 ppm Se | p4 | Gpx3+/+ | wt | 1 | 1.479 | 232 | 156.6 |
|  | 0.25 ppm Se | p5 | Gpx3+/+ | wt | 1 | 1.392 | 232 | 166.7 |
|  | 0.25 ppm Se | p6 | Gpx3+/+ | wt | 1 | 1.283 | 190 | 148.1 |
|  | 0.25 ppm Se | p7 | Gpx3+/+ | wt | 1 | 1.401 | 202 | 144.0 |
|  | 0.25 ppm Se | p8 | Gpx3+/+ | wt | 1 | 1.359 | 202 | 148.9 |
| G3.8-1 | 0.25 ppm Se | p1 | Gpx3+/+ | wt | 1 | 1.323 | 226 | 170.6 |
|  | 0.25 ppm Se | p2 | Gpx3+/+ | wt | 1 | 1.478 | 235 | 159.0 |
|  | 0.25 ppm Se | p3 | Gpx3+/+ | wt | 1 | 1.209 | 208 | 172.3 |
|  | 0.25 ppm Se | p4 | Gpx3+/+ | wt | 1 | 1.365 | 214 | 156.4 |
|  | 0.25 ppm Se | p5 | Gpx3+/+ | wt | 1 | 1.517 | 231 | 152.5 |
|  | 0.25 ppm Se | p6 | Gpx3+/+ | wt | 1 | 1.400 | 216 | 154.4 |
| G3.11-1 | 0.25 ppm Se | p1 | Gpx3+/+ | wt | 1 | 1.430 | 232 | 162.4 |
|  | 0.25 ppm Se | p2 | Gpx3+/+ | wt | 1 | 1.420 | 206 | 144.9 |
|  | 0.25 ppm Se | p3 | Gpx3+/+ | wt | 1 | 1.733 | 282 | 162.6 |
|  | 0.25 ppm Se | p4 | Gpx3+/+ | wt | 1 | 1.429 | 209 | 146.0 |
|  | 0.25 ppm Se | p5 | Gpx3+/+ | wt | 1 | 1.701 | 249 | 146.7 |
| G3.10-2 | 0.25 ppm Se | p1 | Gpx3+/+ | wt | 5 | 2.800 | 424 | 151.5 |
|  | 0.25 ppm Se | p2 | Gpx3+/+ | wt | 5 | 2.800 | 405 | 144.8 |
|  | 0.25 ppm Se | p3 | Gpx3+/+ | wt | 5 | 2.100 | 357 | 169.8 |
|  | 0.25 ppm Se | p4 | Gpx3+/+ | wt | 5 | 2.600 | 401 | 154.1 |
|  | 0.25 ppm Se | p5 | Gpx3+/+ | wt | 5 | 2.970 | 455 | 153.3 |
|  | 0.25 ppm Se | p6 | Gpx3+/+ | wt | 5 | 2.520 | 393 | 156.0 |
|  | 0.25 ppm Se | p7 | Gpx3+/+ | wt | 5 | 2.700 | 401 | 148.5 |
| G3.10-3 | 0.25 ppm Se | p1 | Gpx3+/+ | wt | 5 | 1.950 | 334 | 171.2 |
|  | 0.25 ppm Se | p2 | Gpx3+/+ | wt | 5 | 1.690 | 307 | 181.9 |
|  | 0.25 ppm Se | p3 | Gpx3+/+ | wt | 5 | 2.220 | 367 | 165.3 |
|  | 0.25 ppm Se | p4 | Gpx3+/+ | wt | 5 | 1.600 | 266 | 166.3 |
|  | 0.25 ppm Se | p5 | Gpx3+/+ | wt | 5 | 2.200 | 341 | 154.8 |
|  | 0.25 ppm Se | p6 | Gpx3+/+ | wt | 5 | 2.230 | 380 | 170.4 |
|  | 0.25 ppm Se | p7 | Gpx3+/+ | wt | 5 | 2.230 | 341 | 153.1 |
|  | 0.25 ppm Se | p8 | Gpx3+/+ | wt | 5 | 2.190 | 325 | 148.3 |
|  | 0.25 ppm Se | p9 | Gpx3+/+ | wt | 5 | 2.340 | 375 | 160.0 |
| G3.2-2 | 0.25 ppm Se | p1 | Gpx3+/+ | wt | 5 | 2.460 | 385 | 156.4 |
|  | 0.25 ppm Se | p2 | Gpx3+/+ | wt | 5 | 2.280 | 374 | 164.0 |
|  | 0.25 ppm Se | p3 | Gpx3+/+ | wt | 5 | 2.650 | 390 | 147.3 |
|  | 0.25 ppm Se | p4 | Gpx3+/+ | wt | 5 | 2.800 | 422 | 150.7 |
|  | 0.25 ppm Se | p5 | Gpx3+/+ | wt | 5 | 2.460 | 378 | 153.8 |
|  | 0.25 ppm Se | p6 | Gpx3+/+ | wt | 5 | 2.230 | 359 | 161.0 |
| C57BL/6 #4 | 0 Se | p1 | C57BL/6 | wt | 1 | 1.520 | 35 | 23.0 |
|  | 0 Se | p2 | C57BL/6 | wt | 1 | 1.339 | 29 | 21.8 |
|  | 0 Se | p3 | C57BL/6 | wt | 1 | 1.399 | 29 | 20.4 |
|  | 0 Se | p4 | C57BL/6 | wt | 1 | 1.314 | 25 | 18.7 |
|  | 0 Se | p5 | C57BL/6 | wt | 1 | 1.258 | 26 | 21.0 |
|  | 0 Se | p6 | C57BL/6 | wt | 1 | 1.236 | 27 | 22.1 |
|  | 0 Se | p7 | C57BL/6 | wt | 1 | 1.152 | 22 | 19.0 |
|  | 0 Se | p8 | C57BL/6 | wt | 1 | 1.255 | 27 | 21.9 |
| C57BL/6 #5 | 0 Se | p1 | C57BL/6 | wt | 1 | 1.357 | 26 | 18.8 |
|  | 0 Se | p2 | C57BL/6 | wt | 1 | 1.266 | 25 | 19.9 |
|  | 0 Se | p3 | C57BL/6 | wt | 1 | 1.406 | 28 | 19.7 |
|  | 0 Se | p4 | C57BL/6 | wt | 1 | 1.390 | 30 | 21.5 |
|  | 0 Se | p5 | C57BL/6 | wt | 1 | 1.341 | 20 | 14.6 |
|  | 0 Se | p6 | C57BL/6 | wt | 1 | 1.305 | 28 | 21.8 |
|  | 0 Se | p7 | C57BL/6 | wt | 1 | 1.266 | 32 | 25.0 |
|  | 0 Se | p8 | C57BL/6 | wt | 1 | 1.435 | 39 | 27.4 |
|  | 0 Se | p9 | C57BL/6 | wt | 1 | 1.401 | 34 | 23.9 |
| C57BL/6 #8 | 0 Se | p1 | C57BL/6 | wt | 1 | 1.497 | 18 | 11.7 |
|  | 0 Se | p2 | C57BL/6 | wt | 1 | 1.261 | 22 | 17.4 |
|  | 0 Se | p3 | C57BL/6 | wt | 1 | 1.208 | 18 | 15.1 |
|  | 0 Se | p4 | C57BL/6 | wt | 1 | 1.517 | 25 | 16.3 |
|  | 0 Se | p5 | C57BL/6 | wt | 1 | 1.432 | 24 | 16.5 |
|  | 0 Se | p6 | C57BL/6 | wt | 1 | 1.269 | 15 | 11.6 |
|  | 0 Se | p7 | C57BL/6 | wt | 1 | 1.091 | 17 | 15.2 |
|  | 0 Se | p8 | C57BL/6 | wt | 1 | 1.303 | 23 | 17.8 |
| C57BL/6 #9 | 0 Se | p1 | C57BL/6 | wt | 1 | 1.366 | 18 | 13.2 |
|  | 0 Se | p2 | C57BL/6 | wt | 1 | 1.326 | 20 | 14.9 |
|  | 0 Se | p3 | C57BL/6 | wt | 1 | 1.339 | 24 | 18.2 |
|  | 0 Se | p4 | C57BL/6 | wt | 1 | 1.158 | 7 | 6.4 |
|  | 0 Se | p5 | C57BL/6 | wt | 1 | 1.258 | 17 | 13.6 |
|  | 0 Se | p6 | C57BL/6 | wt | 1 | 0.874 | 19 | 21.3 |
|  | 0 Se | p7 | C57BL/6 | wt | 1 | 1.187 | 15 | 13.0 |
| C57BL/6 #1 | 0 Se | p1 | C57BL/6 | wt | 5 | 1.962 | 59 | 30.0 |
|  | 0 Se | p2 | C57BL/6 | wt | 5 | 1.726 | 48 | 28.0 |
|  | 0 Se | p3 | C57BL/6 | wt | 5 | 1.834 | 46 | 25.4 |
|  | 0 Se | p4 | C57BL/6 | wt | 5 | 1.850 | 53 | 28.9 |
|  | 0 Se | p5 | C57BL/6 | wt | 5 | 1.762 | 46 | 26.3 |
| C57BL/6 #2 | 0 Se | p1 | C57BL/6 | wt | 5 | 2.576 | 73 | 28.3 |
|  | 0 Se | p2 | C57BL/6 | wt | 5 | 2.308 | 69 | 30.0 |
|  | 0 Se | p3 | C57BL/6 | wt | 5 | 1.933 | 61 | 31.6 |
|  | 0 Se | p4 | C57BL/6 | wt | 5 | 2.542 | 71 | 28.0 |
|  | 0 Se | p5 | C57BL/6 | wt | 5 | 2.489 | 65 | 26.1 |
|  | 0 Se | p6 | C57BL/6 | wt | 5 | 2.450 | 51 | 20.9 |
|  | 0 Se | p7 | C57BL/6 | wt | 5 | 2.415 | 54 | 22.5 |
| C57BL/6 #3 | 0 Se | p1 | C57BL/6 | wt | 5 | 2.274 | 50 | 21.8 |
|  | 0 Se | p2 | C57BL/6 | wt | 5 | 2.523 | 62 | 24.4 |
|  | 0 Se | p3 | C57BL/6 | wt | 5 | 2.320 | 50 | 21.4 |
|  | 0 Se | p4 | C57BL/6 | wt | 5 | 2.078 | 47 | 22.4 |
|  | 0 Se | p5 | C57BL/6 | wt | 5 | 2.189 | 31 | 14.2 |
|  | 0 Se | p6 | C57BL/6 | wt | 5 | 2.145 | 27 | 12.4 |
| C57BL/6 #6 | 0 Se | p1 | C57BL/6 | wt | 5 | 2.783 | 38 | 13.5 |
|  | 0 Se | p2 | C57BL/6 | wt | 5 | 2.358 | 40 | 16.8 |
|  | 0 Se | p3 | C57BL/6 | wt | 5 | 2.436 | 34 | 14.1 |
|  | 0 Se | p4 | C57BL/6 | wt | 5 | 2.573 | 42 | 16.5 |
|  | 0 Se | p5 | C57BL/6 | wt | 5 | 2.761 | 44 | 16.0 |
|  | 0 Se | p6 | C57BL/6 | wt | 5 | 2.364 | 21 | 8.7 |
|  | 0 Se | p7 | C57BL/6 | wt | 5 | 2.694 | 36 | 13.3 |
|  | 0 Se | p8 | C57BL/6 | wt | 5 | 2.978 | 48 | 16.1 |
|  | 0 Se | p9 | C57BL/6 | wt | 5 | 2.546 | 46 | 17.9 |
| C57BL/6 #7 | 0 Se | p1 | C57BL/6 | wt | 5 | 3.047 | 42 | 13.8 |
|  | 0 Se | p2 | C57BL/6 | wt | 5 | 3.112 | 42 | 13.4 |
|  | 0 Se | p3 | C57BL/6 | wt | 5 | 2.876 | 41 | 14.1 |
|  | 0 Se | p4 | C57BL/6 | wt | 5 | 3.213 | 37 | 11.6 |
|  | 0 Se | p5 | C57BL/6 | wt | 5 | 3.039 | 38 | 12.6 |
|  | 0 Se | p6 | C57BL/6 | wt | 5 | 2.888 | 35 | 12.1 |
| C57BL/6 #11 | 0.25 ppm Se | p1 | C57BL/6 | wt | 1 | 1.320 | 235 | 178.1 |
|  | 0.25 ppm Se | p2 | C57BL/6 | wt | 1 | 1.420 | 229 | 161.3 |
|  | 0.25 ppm Se | p3 | C57BL/6 | wt | 1 | 1.350 | 215 | 159.1 |
|  | 0.25 ppm Se | p4 | C57BL/6 | wt | 1 | 1.310 | 227 | 173.2 |
|  | 0.25 ppm Se | p5 | C57BL/6 | wt | 1 | 1.210 | 193 | 159.3 |
|  | 0.25 ppm Se | p6 | C57BL/6 | wt | 1 | 1.330 | 217 | 162.8 |
| C57BL/6 #12 | 0.25 ppm Se | p1 | C57BL/6 | wt | 1 | 1.470 | 268 | 182.4 |
|  | 0.25 ppm Se | p2 | C57BL/6 | wt | 1 | 1.370 | 227 | 165.5 |
|  | 0.25 ppm Se | p3 | C57BL/6 | wt | 1 | 1.340 | 227 | 169.5 |
|  | 0.25 ppm Se | p4 | C57BL/6 | wt | 1 | 1.160 | 216 | 186.2 |
|  | 0.25 ppm Se | p5 | C57BL/6 | wt | 1 | 1.130 | 222 | 196.5 |
| C57BL/6 #15 | 0.25 ppm Se | p1 | C57BL/6 | wt | 1 | 1.739 | 275 | 158.1 |
|  | 0.25 ppm Se | p2 | C57BL/6 | wt | 1 | 1.679 | 273 | 162.5 |
|  | 0.25 ppm Se | p3 | C57BL/6 | wt | 1 | 1.656 | 268 | 161.6 |
|  | 0.25 ppm Se | p4 | C57BL/6 | wt | 1 | 1.582 | 251 | 158.9 |
|  | 0.25 ppm Se | p5 | C57BL/6 | wt | 1 | 1.672 | 273 | 163.1 |
| C57BL/6 #17 | 0.25 ppm Se | p1 | C57BL/6 | wt | 1 | 1.411 | 235 | 166.4 |
|  | 0.25 ppm Se | p2 | C57BL/6 | wt | 1 | 1.297 | 210 | 161.8 |
|  | 0.25 ppm Se | p3 | C57BL/6 | wt | 1 | 1.375 | 236 | 171.5 |
|  | 0.25 ppm Se | p4 | C57BL/6 | wt | 1 | 1.437 | 229 | 159.7 |
|  | 0.25 ppm Se | p5 | C57BL/6 | wt | 1 | 1.567 | 255 | 162.4 |
|  | 0.25 ppm Se | p6 | C57BL/6 | wt | 1 | 1.500 | 237 | 157.7 |
|  | 0.25 ppm Se | p7 | C57BL/6 | wt | 1 | 1.445 | 231 | 159.6 |
|  | 0.25 ppm Se | p8 | C57BL/6 | wt | 1 | 1.561 | 235 | 150.3 |
| C57BL/6 #18 | 0.25 ppm Se | p1 | C57BL/6 | wt | 1 | 1.239 | 197 | 159.0 |
|  | 0.25 ppm Se | p2 | C57BL/6 | wt | 1 | 1.327 | 228 | 171.9 |
|  | 0.25 ppm Se | p3 | C57BL/6 | wt | 1 | 1.329 | 212 | 159.7 |
|  | 0.25 ppm Se | p4 | C57BL/6 | wt | 1 | 1.196 | 171 | 143.0 |
|  | 0.25 ppm Se | p5 | C57BL/6 | wt | 1 | 1.301 | 196 | 150.5 |
|  | 0.25 ppm Se | p6 | C57BL/6 | wt | 1 | 1.421 | 218 | 153.2 |
|  | 0.25 ppm Se | p7 | C57BL/6 | wt | 1 | 1.448 | 207 | 143.1 |
|  | 0.25 ppm Se | p8 | C57BL/6 | wt | 1 | 1.449 | 204 | 141.0 |
|  | 0.25 ppm Se | p9 | C57BL/6 | wt | 1 | 1.155 | 160 | 138.6 |
| C57BL/6 #13 | 0.25 ppm Se | p1 | C57BL/7 | wt | 5 | 2.400 | 358 | 149.0 |
|  | 0.25 ppm Se | p2 | C57BL/7 | wt | 5 | 2.060 | 303 | 147.1 |
|  | 0.25 ppm Se | p3 | C57BL/7 | wt | 5 | 2.410 | 351 | 145.7 |
|  | 0.25 ppm Se | p4 | C57BL/7 | wt | 5 | 2.290 | 331 | 144.4 |
|  | 0.25 ppm Se | p5 | C57BL/7 | wt | 5 | 2.140 | 334 | 156.0 |
|  | 0.25 ppm Se | p6 | C57BL/7 | wt | 5 | 2.370 | 356 | 150.0 |
|  | 0.25 ppm Se | p7 | C57BL/7 | wt | 5 | 2.200 | 311 | 141.5 |
| C57BL/6 #14 | 0.25 ppm Se | p1 | C57BL/7 | wt | 5 | 2.475 | 394 | 159.3 |
|  | 0.25 ppm Se | p2 | C57BL/7 | wt | 5 | 2.724 | 418 | 153.5 |
|  | 0.25 ppm Se | p3 | C57BL/7 | wt | 5 | 2.574 | 398 | 154.5 |
|  | 0.25 ppm Se | p4 | C57BL/7 | wt | 5 | 2.568 | 388 | 151.2 |
|  | 0.25 ppm Se | p5 | C57BL/7 | wt | 5 | 2.508 | 374 | 149.2 |
|  | 0.25 ppm Se | p6 | C57BL/7 | wt | 5 | 2.700 | 400 | 148.3 |
|  | 0.25 ppm Se | p7 | C57BL/7 | wt | 5 | 2.471 | 362 | 146.3 |
|  | 0.25 ppm Se | p8 | C57BL/7 | wt | 5 | 1.441 | 248 | 172.4 |
| C57BL/6 #16 | 0.25 ppm Se | p1 | C57BL/7 | wt | 5 | 2.431 | 369 | 151.6 |
|  | 0.25 ppm Se | p2 | C57BL/7 | wt | 5 | 2.139 | 330 | 154.4 |
|  | 0.25 ppm Se | p3 | C57BL/7 | wt | 5 | 2.523 | 378 | 150.0 |
|  | 0.25 ppm Se | p4 | C57BL/7 | wt | 5 | 2.406 | 371 | 154.3 |
|  | 0.25 ppm Se | p5 | C57BL/7 | wt | 5 | 2.109 | 325 | 154.2 |
|  | 0.25 ppm Se | p6 | C57BL/7 | wt | 5 | 2.437 | 357 | 146.5 |
|  | 0.25 ppm Se | p7 | C57BL/7 | wt | 5 | 2.132 | 336 | 157.4 |
|  | 0.25 ppm Se | p8 | C57BL/7 | wt | 5 | 2.428 | 383 | 157.6 |
|  | 0.25 ppm Se | p9 | C57BL/7 | wt | 5 | 2.375 | 345 | 145.2 |
|  | 0.25 ppm Se | p10 | C57BL/7 | wt | 5 | 2.669 | 394 | 147.6 |
