# Supplementary material for: Selenoprotein P Is the Major Selenium Transport Protein in Mouse Milk
Source: PLoS One. 2014 Jul 28;9(7):e103486. doi: 10.1371/journal.pone.0103486 (PMC4113432; doi:10.1371/journal.pone.0103486)
Supplement: Table S2 — Supporting data for Figure 3 . (DOCX) [file pone.0103486.s003.docx]

Table S2 (supporting data for Figure 3)

| dam ID# | diet | dam strain | milk Se (ng/g) |
| --- | --- | --- | --- |
|  |  |  |  |
| 10.756 | 0.25 ppm Se | Sepp1-/- | 83 |
| 10.759 | 0.25 ppm Se | Sepp1-/- | 132 |
| 10.219 | 0.25 ppm Se | Sepp1-/- | 56 |
| 10.254 | 0.25 ppm Se | Sepp1-/- | 62 |
| 10.237 | 0.25 ppm Se | Sepp1-/- | 127 |
| 10.644 | 0.25 ppm Se | Sepp1-/- | 71 |
| 10.734 | 0.25 ppm Se | Sepp1-/- | 81 |
| 10.673 | 0.25 ppm Se | Sepp1-/- | 63 |
| 10.636 | 0.25 ppm Se | Sepp1-/- | 59 |
| 10.717 | 0.25 ppm Se | Sepp1+/+ | 256 |
| 10.771 | 0.25 ppm Se | Sepp1+/+ | 341 |
| 10.762 | 0.25 ppm Se | Sepp1+/+ | 351 |
| 10.228 | 0.25 ppm Se | Sepp1+/+ | 256 |
| G3.14-1 | 0.25 ppm Se | Gpx3-/- | 133 |
| G3.7-2 | 0.25 ppm Se | Gpx3-/- | 114 |
| G3.16-1 | 0.25 ppm Se | Gpx3-/- | 174 |
| G3.13-1 | 0.25 ppm Se | Gpx3-/- | 183 |
| G3.7-3 | 0.25 ppm Se | Gpx3-/- | 193 |
| G3.5-2 | 0.25 ppm Se | Gpx3-/- | 162 |
| G3.15-2 | 0.25 ppm Se | Gpx3-/- | 157 |
| G3.9-1 | 0.25 ppm Se | Gpx3+/+ | 124 |
| G3.11-3 | 0.25 ppm Se | Gpx3+/+ | 149 |
| G3.9-2 | 0.25 ppm Se | Gpx3+/+ | 178 |
| G3.3-3 | 0.25 ppm Se | Gpx3+/+ | 141 |
| G3.3-2 | 0.25 ppm Se | Gpx3+/+ | 114 |
| G3.10-2 | 0.25 ppm Se | Gpx3+/+ | 145 |
