# Supplementary material for: Selenoprotein P Is the Major Selenium Transport Protein in Mouse Milk
Source: PLoS One. 2014 Jul 28;9(7):e103486. doi: 10.1371/journal.pone.0103486 (PMC4113432; doi:10.1371/journal.pone.0103486)
Supplement: Table S3 — Supporting data for Figure 6 . (DOCX) [file pone.0103486.s004.docx]

Table S3 (supporting data for Figure 6)

| dam ID# | diet | neonate ID# | dam strain | neonate genotype | age (day) | whole body Se (ng/g) |
| --- | --- | --- | --- | --- | --- | --- |
| A.854 | 0.25 ppm Se | p1 | apoER2-/- | ht | 1 | 198 |
|  | 0.25 ppm Se | p2 | apoER2-/- | ht | 1 | 201 |
|  | 0.25 ppm Se | p3 | apoER2-/- | ht | 1 | 190 |
|  | 0.25 ppm Se | p4 | apoER2-/- | ht | 1 | 194 |
| A.829 | 0.25 ppm Se | p1 | apoER2-/- | ht | 1 | 162 |
|  | 0.25 ppm Se | p2 | apoER2-/- | ht | 1 | 170 |
|  | 0.25 ppm Se | p3 | apoER2-/- | ht | 1 | 167 |
|  | 0.25 ppm Se | p4 | apoER2-/- | ht | 1 | 160 |
|  | 0.25 ppm Se | p5 | apoER2-/- | ht | 1 | 162 |
|  | 0.25 ppm Se | p6 | apoER2-/- | ht | 1 | 178 |
|  | 0.25 ppm Se | p7 | apoER2-/- | ht | 1 | 167 |
| A.950 | 0.25 ppm Se | p1 | apoER2-/- | ht | 1 | 167 |
|  | 0.25 ppm Se | p2 | apoER2-/- | ht | 1 | 174 |
|  | 0.25 ppm Se | p3 | apoER2-/- | ht | 1 | 166 |
|  | 0.25 ppm Se | p4 | apoER2-/- | ht | 1 | 161 |
|  | 0.25 ppm Se | p5 | apoER2-/- | ht | 1 | 185 |
|  | 0.25 ppm Se | p6 | apoER2-/- | ht | 1 | 183 |
|  | 0.25 ppm Se | p7 | apoER2-/- | ht | 1 | 167 |
|  | 0.25 ppm Se | p8 | apoER2-/- | ht | 1 | 174 |
|  | 0.25 ppm Se | p9 | apoER2-/- | ht | 1 | 177 |
|  | 0.25 ppm Se | p10 | apoER2-/- | ht | 1 | 169 |
|  | 0.25 ppm Se | p11 | apoER2-/- | ht | 1 | 184 |
| A.861 (1st litter) | 0.25 ppm Se | p1 | apoER2-/- | ht | 1 | 193 |
| A.924 | 0.25 ppm Se | p1 | apoER2-/- | ht | 1 | 184 |
|  | 0.25 ppm Se | p2 | apoER2-/- | ht | 1 | 219 |
|  | 0.25 ppm Se | p3 | apoER2-/- | ht | 1 | 195 |
|  | 0.25 ppm Se | p4 | apoER2-/- | ht | 1 | 180 |
|  | 0.25 ppm Se | p5 | apoER2-/- | ht | 1 | 218 |
|  | 0.25 ppm Se | p6 | apoER2-/- | ht | 1 | 195 |
|  | 0.25 ppm Se | p7 | apoER2-/- | ht | 1 | 206 |
| A.861 (2nd litter) | 0.25 ppm Se | p1 | apoER2-/- | ht | 5 | 138 |
|  | 0.25 ppm Se | p2 | apoER2-/- | ht | 5 | 152 |
|  | 0.25 ppm Se | p3 | apoER2-/- | ht | 5 | 151 |
| A.856 (1st litter) | 0.25 ppm Se | p1 | apoER2-/- | ht | 5 | 160 |
|  | 0.25 ppm Se | p2 | apoER2-/- | ht | 5 | 161 |
|  | 0.25 ppm Se | p3 | apoER2-/- | ht | 5 | 155 |
|  | 0.25 ppm Se | p4 | apoER2-/- | ht | 5 | 156 |
| A.856 (2nd litter) | 0.25 ppm Se | p1 | apoER2-/- | ht | 5 | 161 |
|  | 0.25 ppm Se | p2 | apoER2-/- | ht | 5 | 170 |
|  | 0.25 ppm Se | p3 | apoER2-/- | ht | 5 | 160 |
|  | 0.25 ppm Se | p4 | apoER2-/- | ht | 5 | 164 |
|  | 0.25 ppm Se | p5 | apoER2-/- | ht | 5 | 169 |
|  | 0.25 ppm Se | p6 | apoER2-/- | ht | 5 | 171 |
|  | 0.25 ppm Se | p7 | apoER2-/- | ht | 5 | 168 |
|  | 0.25 ppm Se | p8 | apoER2-/- | ht | 5 | 173 |
| A.935 | 0.25 ppm Se | p1 | apoER2-/- | ht | 5 | 162 |
|  | 0.25 ppm Se | p2 | apoER2-/- | ht | 5 | 162 |
|  | 0.25 ppm Se | p3 | apoER2-/- | ht | 5 | 159 |
|  | 0.25 ppm Se | p4 | apoER2-/- | ht | 5 | 157 |
|  | 0.25 ppm Se | p5 | apoER2-/- | ht | 5 | 162 |
|  | 0.25 ppm Se | p6 | apoER2-/- | ht | 5 | 164 |
|  | 0.25 ppm Se | p7 | apoER2-/- | ht | 5 | 153 |
| A.934 | 0.25 ppm Se | p1 | apoER2-/- | ht | 5 | 152 |
|  | 0.25 ppm Se | p2 | apoER2-/- | ht | 5 | 154 |
|  | 0.25 ppm Se | p3 | apoER2-/- | ht | 5 | 149 |
|  | 0.25 ppm Se | p4 | apoER2-/- | ht | 5 | 151 |
|  | 0.25 ppm Se | p5 | apoER2-/- | ht | 5 | 156 |
|  | 0.25 ppm Se | p6 | apoER2-/- | ht | 5 | 145 |
|  | 0.25 ppm Se | p7 | apoER2-/- | ht | 5 | 143 |
| A.925 | 0.25 ppm Se | p1 | apoER2-/- | ht | 5 | 167 |
|  | 0.25 ppm Se | p2 | apoER2-/- | ht | 5 | 171 |
|  | 0.25 ppm Se | p3 | apoER2-/- | ht | 5 | 163 |
|  | 0.25 ppm Se | p4 | apoER2-/- | ht | 5 | 153 |
|  | 0.25 ppm Se | p5 | apoER2-/- | ht | 5 | 159 |
|  | 0.25 ppm Se | p6 | apoER2-/- | ht | 5 | 166 |
| A.958 | 0.25 ppm Se | p1 | apoER2-/- | ht | 5 | 179 |
|  | 0.25 ppm Se | p2 | apoER2-/- | ht | 5 | 178 |
|  | 0.25 ppm Se | p3 | apoER2-/- | ht | 5 | 165 |
|  | 0.25 ppm Se | p4 | apoER2-/- | ht | 5 | 181 |
|  | 0.25 ppm Se | p5 | apoER2-/- | ht | 5 | 169 |
|  | 0.25 ppm Se | p6 | apoER2-/- | ht | 5 | 167 |
|  | 0.25 ppm Se | p7 | apoER2-/- | ht | 5 | 154 |
| A.960 | 0.25 ppm Se | p1 | apoER2+/+ | ht | 1 | 178 |
|  | 0.25 ppm Se | p2 | apoER2+/+ | ht | 1 | 166 |
|  | 0.25 ppm Se | p3 | apoER2+/+ | wt | 1 | 170 |
|  | 0.25 ppm Se | p4 | apoER2+/+ | wt | 1 | 160 |
|  | 0.25 ppm Se | p5 | apoER2+/+ | wt | 1 | 160 |
|  | 0.25 ppm Se | p6 | apoER2+/+ | wt | 1 | 151 |
|  | 0.25 ppm Se | p7 | apoER2+/+ | ht | 1 | 166 |
|  | 0.25 ppm Se | p8 | apoER2+/+ | wt | 1 | 158 |
| A.004 | 0.25 ppm Se | p1 | apoER2+/+ | ht | 1 | 151 |
|  | 0.25 ppm Se | p2 | apoER2+/+ | wt | 1 | 149 |
|  | 0.25 ppm Se | p3 | apoER2+/+ | wt | 1 | 145 |
|  | 0.25 ppm Se | p4 | apoER2+/+ | wt | 1 | 134 |
|  | 0.25 ppm Se | p5 | apoER2+/+ | wt | 1 | 152 |
|  | 0.25 ppm Se | p6 | apoER2+/+ | wt | 1 | 137 |
| A.933 (1st litter) | 0.25 ppm Se | p1 | apoER2+/+ | wt | 1 | 157 |
|  | 0.25 ppm Se | p2 | apoER2+/+ | wt | 1 | 153 |
|  | 0.25 ppm Se | p3 | apoER2+/+ | ht | 1 | 156 |
|  | 0.25 ppm Se | p4 | apoER2+/+ | ht | 1 | 153 |
|  | 0.25 ppm Se | p5 | apoER2+/+ | ht | 1 | 159 |
|  | 0.25 ppm Se | p6 | apoER2+/+ | ht | 1 | 146 |
|  | 0.25 ppm Se | p7 | apoER2+/+ | wt | 1 | 158 |
|  | 0.25 ppm Se | p8 | apoER2+/+ | wt | 1 | 134 |
| A.974 | 0.25 ppm Se | p1 | apoER2+/+ | wt | 1 | 179 |
|  | 0.25 ppm Se | p2 | apoER2+/+ | ht | 1 | 187 |
|  | 0.25 ppm Se | p3 | apoER2+/+ | wt | 1 | 170 |
|  | 0.25 ppm Se | p4 | apoER2+/+ | wt | 1 | 180 |
|  | 0.25 ppm Se | p5 | apoER2+/+ | ht | 1 | 170 |
|  | 0.25 ppm Se | p6 | apoER2+/+ | ht | 1 | 161 |
| A.932 | 0.25 ppm Se | p1 | apoER2+/+ | wt | 5 | 168 |
|  | 0.25 ppm Se | p2 | apoER2+/+ | wt | 5 | 184 |
|  | 0.25 ppm Se | p3 | apoER2+/+ | ht | 5 | 167 |
|  | 0.25 ppm Se | p4 | apoER2+/+ | wt | 5 | 165 |
|  | 0.25 ppm Se | p5 | apoER2+/+ | ht | 5 | 167 |
|  | 0.25 ppm Se | p6 | apoER2+/+ | ht | 5 | 165 |
| A.933 (2nd litter) | 0.25 ppm Se | p1 | apoER2+/+ | wt | 5 | 149 |
|  | 0.25 ppm Se | p2 | apoER2+/+ | ht | 5 | 149 |
|  | 0.25 ppm Se | p3 | apoER2+/+ | wt | 5 | 146 |
|  | 0.25 ppm Se | p4 | apoER2+/+ | wt | 5 | 146 |
|  | 0.25 ppm Se | p5 | apoER2+/+ | ht | 5 | 147 |
|  | 0.25 ppm Se | p6 | apoER2+/+ | wt | 5 | 139 |
|  | 0.25 ppm Se | p7 | apoER2+/+ | wt | 5 | 143 |
| A.960 | 0.25 ppm Se | p1 | apoER2+/+ | ht | 5 | 176 |
|  | 0.25 ppm Se | p2 | apoER2+/+ | wt | 5 | 163 |
|  | 0.25 ppm Se | p3 | apoER2+/+ | ht | 5 | 160 |
|  | 0.25 ppm Se | p4 | apoER2+/+ | ht | 5 | 151 |
|  | 0.25 ppm Se | p5 | apoER2+/+ | wt | 5 | 156 |
| A.983 | 0.25 ppm Se | p1 | apoER2+/+ | wt | 5 | 161 |
|  | 0.25 ppm Se | p2 | apoER2+/+ | ht | 5 | 170 |
|  | 0.25 ppm Se | p3 | apoER2+/+ | ht | 5 | 155 |
|  | 0.25 ppm Se | p4 | apoER2+/+ | wt | 5 | 151 |
|  | 0.25 ppm Se | p5 | apoER2+/+ | ht | 5 | 155 |
|  | 0.25 ppm Se | p6 | apoER2+/+ | wt | 5 | 154 |
| A.959 | 0.25 ppm Se | p1 | apoER2+/+ | ht | 5 | 156 |
|  | 0.25 ppm Se | p2 | apoER2+/+ | wt | 5 | 158 |
|  | 0.25 ppm Se | p3 | apoER2+/+ | ht | 5 | 163 |
|  | 0.25 ppm Se | p4 | apoER2+/+ | ht | 5 | 164 |
|  | 0.25 ppm Se | p5 | apoER2+/+ | ht | 5 | 155 |
